# Differential gene expression of bone anabolic factors and trabecular bone architectural changes in the proximal femoral shaft of primary hip osteoarthritis patients

**DOI:** 10.1186/ar2101

**Published:** 2006-12-22

**Authors:** Le-Hoa Truong, Julia S Kuliwaba, Helen Tsangari, Nicola L Fazzalari

**Affiliations:** 1 Bone and Joint Research Laboratory, Division of Tissue Pathology, Institute of Medical and Veterinary Science and the Hanson Institute, Frome Road, Adelaide, 5000, Australia; 2 Discipline of Pathology, School of Medical Sciences, The University of Adelaide, Frome Road, Adelaide, 5005, Australia

## Abstract

Previous studies have shown a generalised increase in bone mass in patients with osteoarthritis (OA). Using molecular histomorphometry, this study examined the *in vivo *expression of mRNA encoding bone anabolic factors and collagen type I genes (*COL1A1*, *COL1A2*) in human OA and non-OA bone. Bone samples were obtained from the intertrochanteric (IT) region of the proximal femur, a skeletal site distal to the active site of disease, from individuals with hip OA at joint replacement surgery and from autopsy controls. Semi-quantitative reverse transcription-polymerase chain reaction analysis revealed elevated mRNA expression levels of alkaline phosphatase (*p *< 0.002), osteocalcin (OCN) (*p *< 0.0001), osteopontin (*p *< 0.05), COL1A1 (*p *< 0.0001), and COL1A2 (*p *< 0.002) in OA bone compared to control, suggesting possible increases in osteoblastic biosynthetic activity and/or bone turnover at the IT region in OA. Interestingly, the ratio of COL1A1/COL1A2 mRNA was almost twofold greater in OA bone compared to control (*p *< 0.001), suggesting the potential presence of collagen type I homotrimer at the distal site. Insulin-like growth factor (IGF)-I, IGF-II, and transforming growth factor-β1 mRNA levels were similar between OA and control bone. Bone histomorphometric analysis indicated that OA IT bone had increased surface density of bone (*p *< 0.0003), increased trabecular number (Tb.N) (*p *< 0.0003), and decreased trabecular separation (Tb.Sp) (*p *< 0.0001) compared to control bone. When the molecular and histomorphometric data were plotted, positive associations were observed in the controls for OCN/glyceraldehyde-3-phosphate dehydrogenase (GAPDH) versus bone tissue volume (*r *= 0.82, *p *< 0.0007) and OCN/GAPDH versus Tb.N (*r *= 0.56, *p *< 0.05) and a negative association was observed for OCN/GAPDH versus Tb.Sp (*r *= -0.64, *p *< 0.02). These relationships were not evident in trabecular bone from patients with OA, suggesting that bone regulatory processes leading to particular trabecular structures may be altered in this disease. The finding of differential gene expression, as well as architectural changes and differences in molecular histomorphometric associations between OA and controls, at a skeletal site distal to the active site of joint degeneration supports the concept of generalised involvement of bone in the pathogenesis of OA.

## Introduction

Osteoarthritis (OA) is an age-related degenerative musculoskeletal disease affecting both males and females and causing significant morbidity and immobility. OA is characterised by loss of articular cartilage, subchondral bone architectural changes, and altered joint biomechanical and biochemical properties, which may be contributed to by environmental and genetic influences [1]. The pathogenesis of OA is still unknown.

Accumulating evidence supports the hypothesis that OA is a bone disease instead of or in addition to a cartilage disease [2]. There is substantial evidence from spontaneous OA animal models of a change in the density and metabolism of subchondral bone prior to any signs of cartilage damage (reviewed in [2,3]). Human OA subchondral bone is sclerotic yet mechanically weak due to hypomineralisation, increased collagen metabolism, and altered bone remodelling [2,4-7]. An increased secretion of type I collagen homotrimer from cultured subchondral osteoblast (OB) cells may contribute to the hypomineralisation in OA bone [8]. The ability of collagen to provide a strong network and to fully mineralise depends on the precise alignment of the type I collagen molecules in the collagen fibre. With the presence of type I collagen homotrimer, collagen fibres have been observed to be narrower and aligned in a disorganised manner in OA subchondral bone [8]. The OA bone changes observed at the subchondral region are also present at skeletal sites distal to the active joint articular cartilage degeneration, such as the intertrochanteric (IT) and medial principal compressive regions of the proximal femur and iliac crest. Studies investigating these distal skeletal sites have found evidence of increased bone volume and trabecular thickness (Tb.Th) and decreased trabecular separation (Tb.Sp) in OA compared to non-OA individuals [9-12]. These compositional and architectural alterations in OA bone reflect differences in bone metabolism and remodelling compared to normal bone physiology. A number of bone-related factors, such as osteocalcin (OCN) and alkaline phosphatase (ALP), both of which are commonly used as markers of bone formation, have been shown to be differentially expressed in OA serum, *in vitro*, and *ex vivo *disease studies [6,13-17]. The finding of altered bone anabolic factor expression levels between normal and OA bone suggests abnormal bone cell behaviour in OA [15,18]. Specifically, cultured OB cells from OA subchondral bone have been shown to be capable of influencing cartilage metabolism [19] and to have markedly altered phenotypic characteristics [15]. The OA OB-like cells in culture are more biosynthetically active, producing increased protein levels of ALP, OCN, and insulin-like growth factor (IGF)-I [15]. These OB-cell phenotypic and functional differences may play an important role in the regulation of bone remodelling in OA individuals.

Patients with primary or idiopathic OA of the hip have been observed to have a higher bone mineral density (BMD) at local and distal skeletal sites [20-22], suggesting generalised skeletal differences in OA individuals which are not necessarily secondary to joint cartilage degeneration. A recent study indicated that high-level hip and spinal BMD measurements at baseline were associated with increased incidence and progression of knee OA, after adjustments for body mass index, age, and gender [23]. Also, the mRNA expression level of regulators of osteoclastogenesis and catabolic factors at the IT region of the proximal femur is decreased; consistent with this, further histomorphometric analyses found decreased resorbing surfaces and increased bone formation relative to bone resorption in patients with primary hip OA [24]. The molecular factors controlling the increase in bone formation at a distal skeletal site in hip OA are yet to be fully understood.

This study used molecular histomorphometry to investigate gene expression of a select group of bone anabolic factors, as well as alpha chains corresponding to collagen type I, at a skeletal site distal to the active site of disease in patients with primary hip OA compared to non-OA controls. In addition, associations between gene expression and bone architecture were explored. The results of the data are indicative of the generalised skeletal distribution of primary OA pathology and suggest that gene expression level differences may influence trabecular architectural changes that ultimately lead to altered bone biomechanical and biochemical properties that, in turn, lead to susceptibility for the progression of joint articular cartilage degeneration.

## Materials and methods

### Human bone specimens

**Figure 1:**
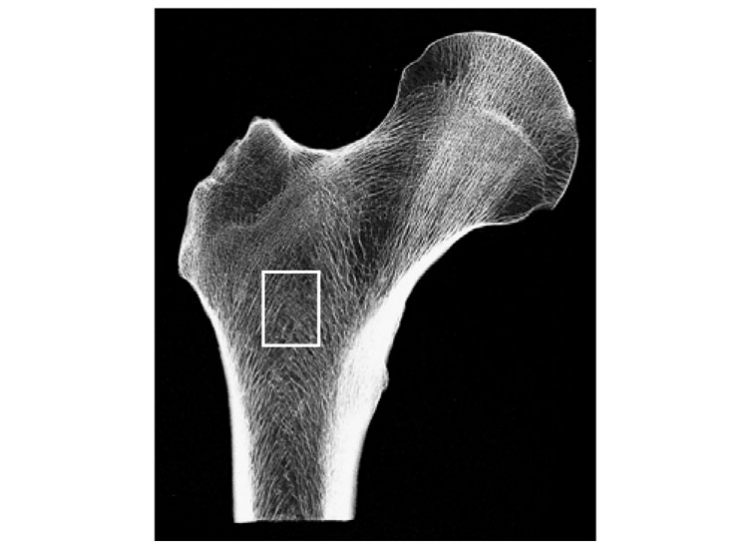
X-ray of a normal proximal femur, showing the intertrochanteric region (rectangle) used for sampling.

A 10-mm tube saw bone biopsy of approximately 30 mm in length from the IT region of the proximal femur (Figure 1) was obtained from 15 patients with primary hip OA (8 females, 48 to 82 years old, and 7 males, 50 to 85 years old; mean age = 65.1 ± 12.6 standard deviation [SD] years) undergoing total hip arthroplasty surgery. The closely age- and gender-matched control group, for which trabecular bone from the same site was taken, comprised 13 autopsy cases (6 females, 57 to 83 years old, and 7 males, 44 to 71 years old; mean age = 63.5 ± 11.2 years) known not to have suffered from any chronic condition or disease that may have affected the skeleton. For both the OA and control groups, cases with a known history of medication that may have affected bone metabolism were excluded. The mean age of the OA group did not differ significantly from that of the control group.

The surgical and autopsy femoral heads were macroscopically graded for OA according to the criteria of Collins [25], as previously described [16,24]. At surgery, primary OA femoral heads were either grade III or IV and the graded autopsy femoral heads were not worse than grade II OA. The use of the IT region of the proximal femur chosen for sampling has been justified previously [24]. Furthermore, architectural and catabolic gene expression differences have been observed between OA and non-OA individuals at this distal skeletal site [12,16,24]. Each trabecular IT bone specimen was divided lengthwise for molecular and histology analyses. Informed consent was obtained for the collection and use of bone specimens, with approval by the Royal Adelaide Hospital Research Ethics Committee.

### Total RNA extraction

For total RNA extraction, the fresh surgical IT bone specimens (stored at 4°C up to 12 hours in sterile RNase-free phosphate-buffered saline) and control bone (obtained 24 to 96 hours after death) were rinsed briefly in diethylpyrocarbonate-treated water (Sigma-Aldrich, St. Louis, MO, USA) and then separated into small fragments by using bone cutters. High-quality total RNA was isolated using a modified guanidinium thiocyanate method of Chomczynski and Sacchi [26], as previously detailed [16,24,27,28]. The procedure for specimen storage, handling, and use of RNA extracted from the IT region from both OA and autopsy controls has been validated by Kuliwaba and colleagues [16,27]. Total RNA extracted from cultured human OA OB cells, obtained from trabecular bone explants [29], served as positive controls for the subsequent semi-quantitative reverse transcription-polymerase chain reaction (SQRT-PCR). The quality and integrity of the RNA extracted were confirmed on 1% wt/vol ethidium bromide-stained formaldehyde-agarose gels.

### Semi-quantitative reverse transcription-polymerase chain reaction

First-strand cDNA synthesis of 1 μg of total RNA isolated from the OA and control bone samples was prepared using a cDNA synthesis kit, Superscript II (Invitrogen Corporation, Carlsbad, CA, USA) with 250 ng of random hexamer primer (GeneWorks Pty Ltd, Adelaide, Australia) according to the manufacturer's instructions. cDNA was synthesised from RNA samples of each group at the same time to limit differences in the efficiency of the cDNA synthesis. Synthesised cDNA was amplified by PCR using mRNA-specific primers to generate products corresponding to mRNA encoding human ALP, collagen type I alpha chain (COL1A) 1, COL1A2, IGF-I, IGF-II, OCN, osteopontin (OPN), transforming growth factor-β1 (TGF-β1), and the housekeeping gene glyceraldehyde-3-phosphate dehydrogenase (*GAPDH*) using reaction mixtures and conditions as previously detailed [16,24,27,30]. Amplification of GAPDH served as an internal positive control and allowed normalisation of the various mRNA levels against the total mRNA content in the samples. The human mRNA-specific primer sequences, predicted PCR product sizes, and optimised PCR conditions are presented in Table 1. To allow semi-quantitation of the PCR products, preliminary experiments were performed to ensure that the PCR cycles were within the exponential phase of the amplification curve. As previously reported in Tsangari and colleagues [28], results obtained by the SQRT-PCR method are comparable to the results obtained using the quantitative Taqman (Applied Biosystems, Foster City, CA, USA) PCR system. Amplifications of each mRNA species for both the OA and control cases were visualised on a single 2% wt/vol agarose gel post-stained with SYBR^® ^Gold (catalog no. S11494; Molecular Probes Inc., now part of Invitrogen Corporation) to minimise interassay variability and were quantitated using the FluorImager/Typhoon and ImageQuant software (Molecular Dynamics, now part of GE Healthcare, Little Chalfont, Buckinghamshire, UK), as previously described [16,24,27].

### Bone histomorphometry

For histology, trabecular bone samples were fixed in 70% ethanol, processed undecalcified through a graded series of ethanol, embedded in methylmethacrylate resin, and sectioned on a microtome (Polycut-E, Leica SP2600; Leica Microsystems, Wetzlar, Germany), as previously described [24,31]. There was insufficient bone tissue for histology for one female OA case (82 years old). Sections, 5 μm thick, were stained by the von Kossa silver method and counterstained with haematoxylin and eosin to distinguish between the mineralised bone, the osteoid, and the cellular components of the marrow. Bone histomorphometric analysis was performed using an ocular-mounted 10 × 10 graticule at a magnification of ×100. Measurements were made of the following parameters: bone tissue volume (BV/TV) (percentage), bone surface density (BS/TV) (square millimetres per cubic millimetre), specific surface of bone (BS/BV) (square millimetres per cubic millimetre), Tb.Th (micrometre), Tb.Sp (micrometre), trabecular number (Tb.N) (number per millimetre), osteoid volume (OV/TV) (percentage), osteoid surface (OS/BS) (percentage), and eroded surface (ES/BS) (percentage). It is worth noting that Tb.N is a derived index of BS/TV and hence will have similar distribution and significance level of data for both OA and control groups.

### Data analysis

The data generated were tested for normality using the Shapiro-Wilk statistic. The statistical significance of the difference between the OA and the control group was determined by Student's *t *test for normally distributed data, expressed as mean ± SD, and Mann-Whitney *U *test for non-parametric data, expressed as median (25th percentile to 75th percentile). Regression analysis using parametric Pearson (*r*) statistics was used to examine age-related changes, the relationship between PCR product/GAPDH ratios and between PCR product/GAPDH ratios, and bone histomorphometric variables (PC-SAS statistical software; SAS Institute Inc., Cary, NC, USA). The critical value for significance was chosen as *p *less than 0.05.

## Results

**Figure 2:**
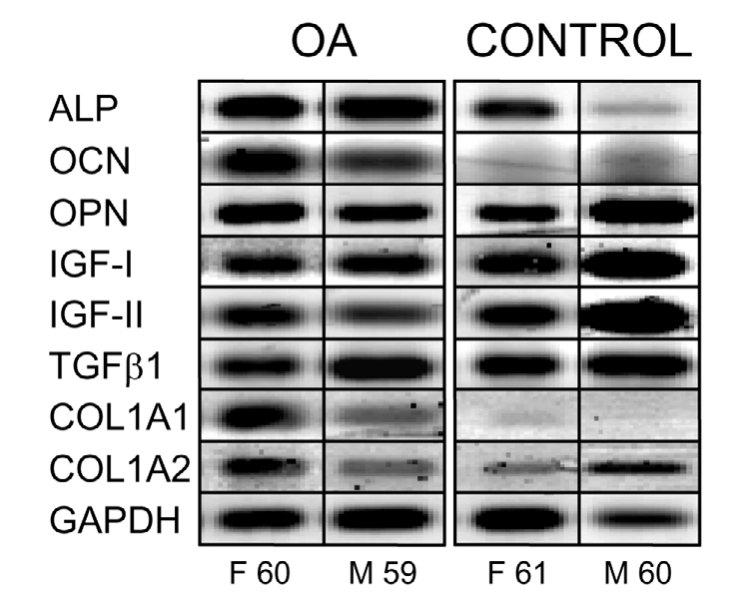
Representative gene expression as determined by semi-quantitative reverse transcription-polymerase chain reaction (PCR) using total RNA extracted from intertrochanteric trabecular bone. Target genes included alkaline phosphatase (*ALP*) (434 bp), osteocalcin (*OCN*) (159 bp), osteopontin (*OPN*) (472 bp), insulin-like growth factor (*IGF*)-I (344 bp), *IGF-II *(263 bp), transforming growth factor-β1 (*TGF-β1*) (224 bp), *COL1A1 *(339 bp), *COL1A2 *(778 bp), and the housekeeping gene *GAPDH *(415 bp). Specimens were obtained from a 60-year-old female (F 60) and a 59-year-old male (M 59) undergoing total hip replacement for primary osteoarthritis (OA). The control specimens were obtained at autopsy from a 61-year-old female (F 61) and a 60-year-old male (M 60) without any bone-related disease. PCR products representing each mRNA species were visualised on SYBR Gold^®^-stained 2% agarose gels. COL1A, collagen type I alpha chain; GAPDH, glyceraldehyde-3-phosphate dehydrogenase.

mRNA corresponding to each of the targeted genes was found to be expressed in trabecular bone from the IT region of the proximal femur for both OA and control individuals (relative expression in OA and control is presented in Figure 2).

### Gender differences in gene expression and histomorphometric parameters

Few gene expression and histomorphometric differences were observed between males and females when the OA and control groups were analysed separately. TGF-β1/GAPDH was the only significant gene expression difference found between the genders, with OA males having higher gene expression levels than OA females (0.91 ± 0.24 versus 0.64 ± 0.07, respectively; *p *< 0.03). In comparison to the respective female group, OA males (118.8 ± 40.1 μm versus 78.2 ± 26.2 μm, respectively; *p *< 0.05) and control males (130 ± 30 μm versus 90 ± 30 μm, respectively; *p *< 0.03) had increased Tb.Th. Due to the few differences observed, further analyses of the data determining relationships between gene expression and histomorphometric indices were made independent of gender for the OA and control groups.

### Bone anabolic and collagen type I mRNA expression between OA and control individuals

**Figure 3:**
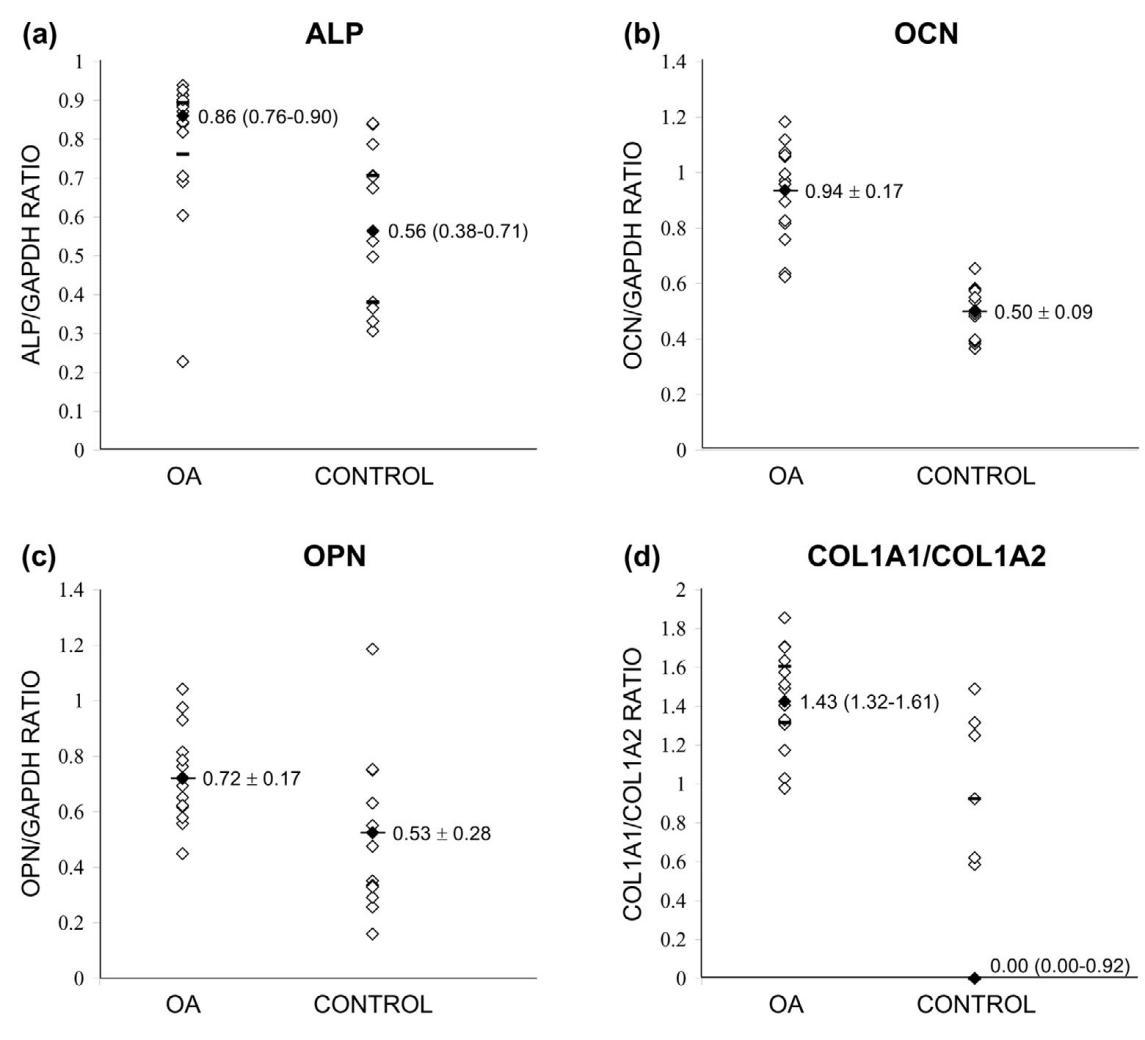
Relative polymerase chain reaction product/GAPDH ratios for alkaline phosphatase (ALP), osteocalcin (OCN), and osteopontin (OPN) and the relative ratio of COL1A1/COL1A2. mRNA expression in intertrochanteric trabecular bone was compared between the osteoarthritis (OA) (*n *= 15) and control (*n *= 13) groups. Patients with OA had significantly elevated **(a) **ALP/GAPDH (*p *< 0.002), **(b) **OCN/GAPDH (*p *< 0.0001), **(c) **OPN/GAPDH (*p *< 0.05), and **(d) **COL1A1/COL1A2 (*p *< 0.0001) mRNA ratios versus controls. Data are expressed as parametric mean ± standard deviation (open diamond) and non-parametric median (closed diamond) and quartile (dash) range. COL1A, collagen type I alpha chain; GAPDH, glyceraldehyde-3-phosphate dehydrogenase.

mRNA corresponding to ALP and OCN, both commonly used as markers of bone formation, and the non-collagenous protein OPN were significantly elevated in the OA group in comparison with the controls (*p *< 0.002, *p *< 0.0001, and *p *< 0.05, respectively; Figure 3a–c). In contrast, IGF-I/GAPDH, IGF-II/GAPDH, and TGF-β1/GAPDH growth factor gene expressions were similar between OA and control individuals. The mean and median values for the growth factors, as well as the differential gene expression of the α1 and α2 chain of collagen type I (*p *< 0.0001 and *p *< 0.002, respectively) between OA and controls, are shown in Table 2. Interestingly, the ratio of COL1A1/COL1A2 was significantly greater in OA bone compared to control (*p *< 0.0001; Figure 3d).

**Figure 4:**
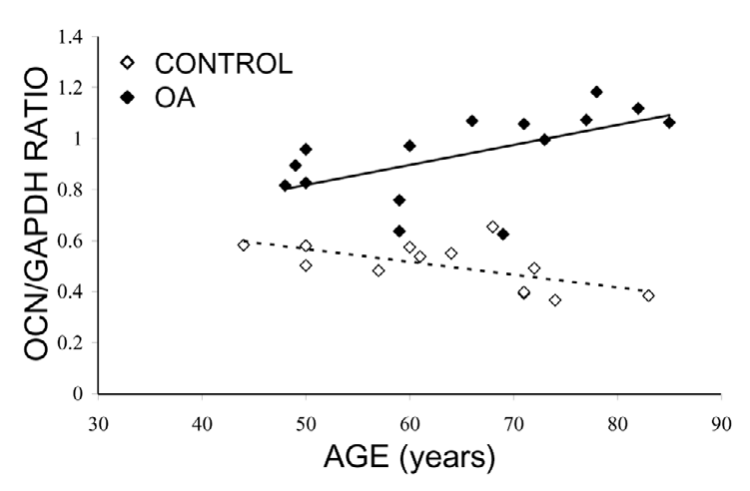
Changes in osteocalcin (OCN)/GAPDH mRNA with age. The relative OCN/GAPDH ratios were determined in intertrochanteric trabecular bone from individuals with osteoarthritis (OA) (*n *= 15) and control individuals (*n *= 13). In OA, OCN/GAPDH mRNA increased significantly with age (OCN/GAPDH = 0.01 [Age] + 0.43; *r *= 0.57, *p *< 0.03). In controls, OCN/GAPDH mRNA significantly declined with age (OCN/GAPDH = -0.01 [Age] + 0.82; *r *= -0.62, *p *< 0.03). GAPDH, glyceraldehyde-3-phosphate dehydrogenase.

An age-related increase in OCN/GAPDH mRNA in OA (*r *= 0.57, *p *< 0.03) and an age-related decrease in controls (*r *= -0.62, *p *< 0.03) were observed (Figure 4). A negative association with age was found for COL1A2/GAPDH mRNA in the OA group only (*n *= 15, *r *= -0.55, *p *< 0.04; data not shown). No other significant age associations were found for the other genes of interest in the two groups.

**Figure 5:**
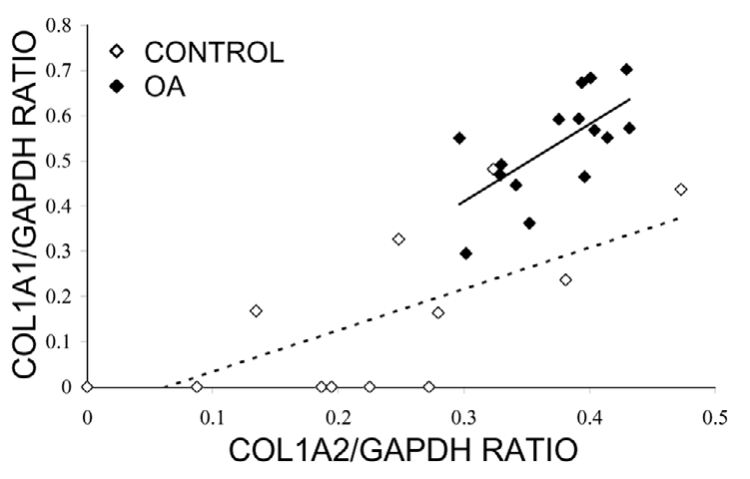
Association between the relative ratios of COL1A1/GAPDH mRNA and COL1A2/GAPDH mRNA. Gene expression was determined in intertrochanteric trabecular bone from patients with osteoarthritis (OA) (*n *= 15) and controls (*n *= 13). A significant correlation was observed between the two parameters in patients with OA (COL1A1/GAPDH = 1.71 [COL1A2/GAPDH] – 0.10; *r *= 0.66, *p *< 0.008) and controls (COL1A1/GAPDH = 0.91 [COL1A2/GAPDH] – 0.06; *r *= 0.70, *p *< 0.008). COL1A, collagen type I alpha chain; GAPDH, glyceraldehyde-3-phosphate dehydrogenase.

Because collagen type I consists of two α1 chains and one α2 chain, the positive association between the two alpha chains observed in both the OA and control groups was expected (*r *= 0.66, *p *< 0.008 and *r *= 0.70, *p *< 0.008, respectively; Figure 5). The slope of the regression line for the OA samples was greater than the slope for the control samples (*p *< 0.001), such that for a given level of COL1A2 mRNA, the corresponding level of COL1A1 mRNA was greater in OA samples. Per unit of COL1A2 gene expression, the level of COL1A1 gene expression in OA was almost double that in the controls (1.71 versus 0.91, respectively).

**Figure 6:**
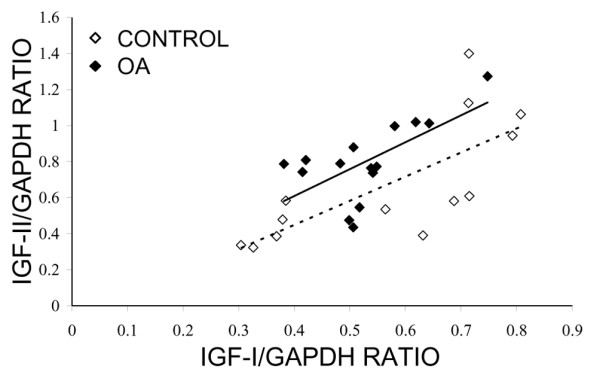
Association between the relative ratios of insulin-like growth factor (IGF)-II/GAPDH mRNA and IGF-I/GAPDH mRNA. Gene expression was determined in intertrochanteric trabecular bone from patients with osteoarthritis (OA) (*n *= 15) and controls (*n *= 13). A significant correlation was observed between the two parameters in patients with OA (IGF-II/GAPDH = 1.49 [IGF-I/GAPDH] + 0.01; *r *= 0.64, *p *< 0.02) and controls (IGF-II/GAPDH = 1.34 [IGF-I/GAPDH] – 0.09; *r *= 0.73, *p *< 0.005). GAPDH, glyceraldehyde-3-phosphate dehydrogenase.

In comparison to the controls, IGF-II/GAPDH mRNA expression was significantly higher than IGF-I/GAPDH mRNA (*p *< 0.0003; Table 2) in the OA group. When the IGF-I/GAPDH mRNA and IGF-II/GAPDH mRNA data were plotted, a significant positive association was observed for both OA and controls (*r *= 0.64, *p *< 0.02 and *r *= 0.73, *p *< 0.005, respectively; Figure 6).

### Comparison of bone structural and turnover indices between OA and control individuals

Bone histomorphometry was performed on IT trabecular bone samples obtained from 14 out of the 15 OA cases from total hip replacement (tissue sample size in one case was insufficient) and the 13 controls without evidence of OA pathology taken at autopsy. The mean and median values for the structural and bone turnover parameters at this skeletal site are shown in Table 3. OA bone had significantly increased BS/TV (*p *< 0.0003) and Tb.N (*p *< 0.0003) and a significant decrease in Tb.Sp (*p *< 0.0001). The static indices for bone formation (OS/BS) and bone resorption (ES/BS) were similar between the OA and control groups. A positive correlation was found between OS/BS and ES/BS for both groups (OA: *n *= 14, OS/BS = 0.97 [ES/BS] + 3.26; *r *= 0.57, *p *< 0.04; control: *n *= 13, OS/BS = 0.56 [ES/BS] + 4.01; *r *= 0.62, *p *< 0.03). This finding suggests that the bone remodelling process is still coupled in the two groups, consistent with previously reported data from the IT region [24].

When the histomorphometric measurements were plotted with age, there was a significant increase in BS/BV (*n *= 13, BS/BV = 0.49 [Age] – 10.7; *r *= 0.66, *p *< 0.02) and a significant decrease in Tb.Th with age for the controls (*n *= 13, Tb.Th = -1.8 [Age] + 223.4; *r *= -0.58, *p *< 0.04). These relationships were not observed for the OA group. Even though there was no significant difference in OS/BS and ES/BS between the OA and control groups, there was a significant association for both of these parameters with age in the controls (*n *= 13, OS/BS = 0.22 [Age] – 6.7; *r *= 0.58, *p *< 0.04 and *n *= 13, ES/BS = 0.29 [Age] – 12.7; *r *= 0.69, *p *< 0.01, respectively), which is consistent with our previous findings [24]. This indicated an increased extent of both bone formation and bone resorption with age, which together suggest an increase in the rate of bone turnover with ageing in control individuals. There were no significant correlations with age for OS/BS or ES/BS in OA.

### Associations between OB marker gene expression and histomorphometry

**Figure 7:**
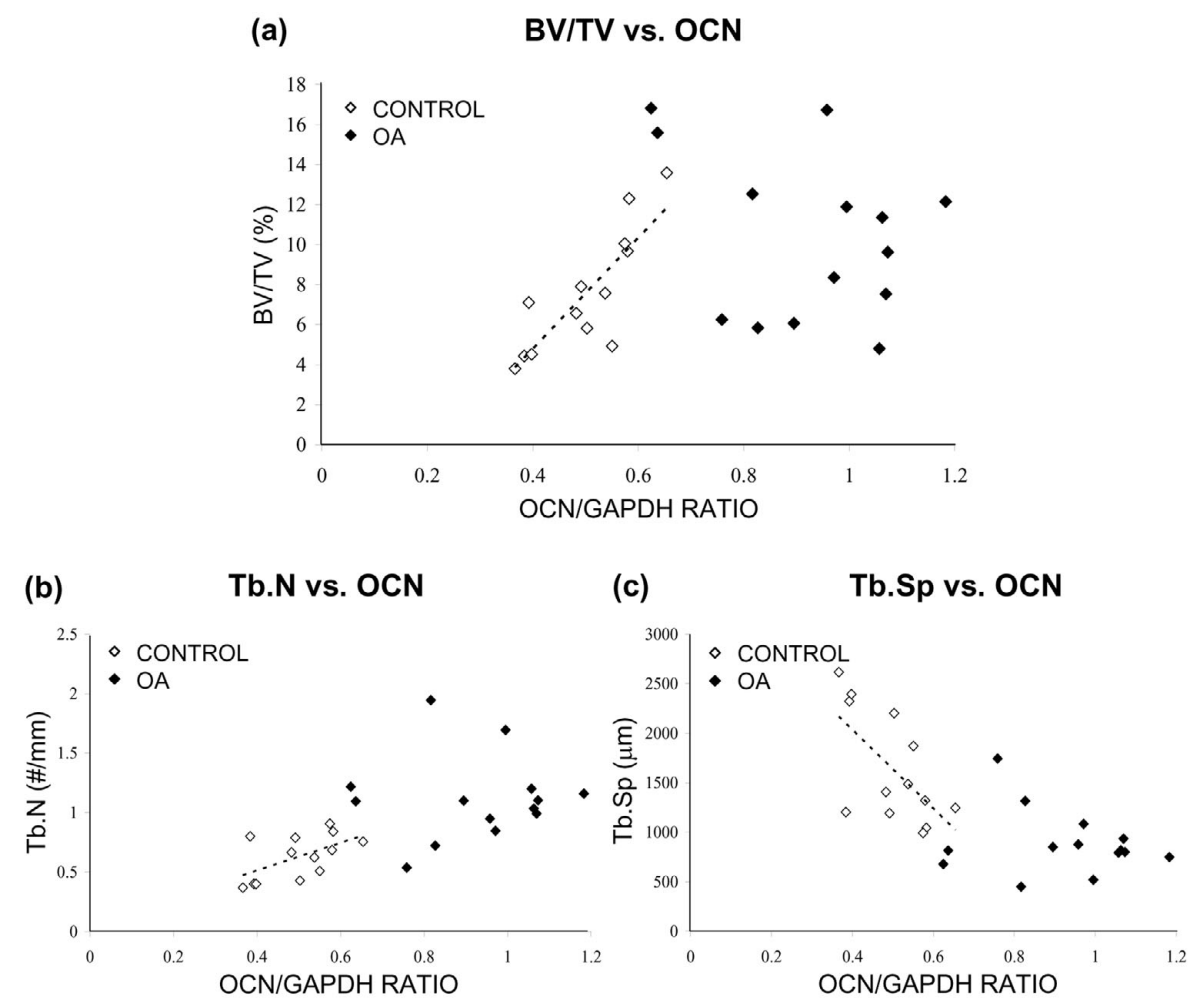
Associations between osteocalcin (OCN)/GAPDH mRNA and the histomorphometric parameters of bone tissue volume (BV/TV), trabecular number (Tb.N), and trabecular separation (Tb.Sp). The relative OCN/GAPDH mRNA expression and architectural parameters were determined in intertrochanteric trabecular bone from osteoarthritis (OA) (*n *= 14) and control (*n *= 13) individuals. **(a) **In controls, there was a significant increase in BV/TV with increasing OCN/GAPDH mRNA (BV/TV = 27.7 [OCN/GAPDH] – 6.3; *r *= 0.82, *p *< 0.0007) in contrast to the patients with OA (BV/TV = -7.57 [OCN/GAPDH] + 17.38; *r *= -0.31, *p *= not significant [NS]). **(b) **A significant increase in Tb.N with increasing OCN/GAPDH mRNA was observed in controls (Tb.N = 1.16 [OCN/GAPDH] + 0.05; *r *= 0.56, *p *< 0.05). In OA, there was no significant association between Tb.N and OCN/GAPDH mRNA (Tb.N = 0.09 [OCN/GAPDH] + 1.03; *r *= 0.04, *p *= NS). **(c) **In controls, there was a significant decline in Tb.Sp with increasing OCN/GAPDH mRNA (Tb.Sp = -3,977.9 [OCN/GAPDH] + 3,626.1; *r *= -0.64, *p *< 0.02) and no significant change in Tb.Sp with OCN/GAPDH mRNA in OA individuals (Tb.Sp = -353.5 [OCN/GAPDH] + 1,214.4; *r *= -0.19, *p *= NS). GAPDH, glyceraldehyde-3-phosphate dehydrogenase.

For the control group, OCN/GAPDH mRNA was found to have a significant positive association with BV/TV (*r *= 0.82, *p *< 0.0007; Figure 7a), BS/TV (*r *= 0.56, *p *< 0.05; data not shown), and Tb.N (*r *= 0.56, *p *< 0.05; Figure 7b) and a significant negative association with Tb.Sp (*r *= -0.64, *p *< 0.02; Figure 7c). These relationships were not observed for the OA group. However, it is interesting to note that the OA data for Tb.N and Tb.Sp had segregated away from the controls such that OA individuals have significantly elevated OCN gene expression with increased Tb.N and decreased Tb.Sp, as reflected in the group comparisons (Figure 3 and Table 3). Furthermore, when the control and OA data were combined for the Tb.N versus OCN/GAPDH (*n *= 27; Tb.N = 0.72 [OCN/GAPDH] + 0.33; *r *= 0.62, *p *< 0.0007) and Tb.Sp versus OCN/GAPDH (*n *= 27; Tb.Sp = 0.91 [OCN/GAPDH] – 0.7; *r *= -0.73, *p *< 0.0001) plots, it appears that there is a continuum in the bone remodelling processes leading to particular trabecular structures, with respect to OCN/GAPDH levels in the IT region. In contrast, ALP, another OB-specific marker, showed a significant decrease in BV/TV (*n *= 13; BV/TV = -9.7 [ALP/GAPDH] + 13.2; *r *= -0.61, *p *< 0.03), BS/TV (*n *= 13; BS/TV = -1.08 [ALP/GAPDH] + 1.88; *r *= -0.56, *p *< 0.05), and Tb.N (*n *= 13; Tb.N = -0.54 [ALP/GAPDH] + 0.9; *r *= -0.56, *p *< 0.05), with increasing ALP/GAPDH mRNA expression in the controls (data not shown). No significant associations were found for OA individuals.

## Discussion

Previous studies have reported higher BMD levels in patients with early- to late-stage OA. However, there is limited knowledge about the molecular and cellular mechanisms involved in the increase or maintenance of bone mass in OA. This study of the IT region of the proximal femur in primary hip OA and non-OA postmortem controls investigated changes in gene expression of bone anabolic factors and collagen type I alpha chains and associations between gene expression and bone micro-architecture.

The bone anabolic factors investigated in this study are known to be involved in the bone remodelling process and there is evidence for their involvement in OA disease [6,15,17,18,24,32]. The results of this study indicated significant elevation in the OB markers, OCN and ALP, as well as OPN and the alpha chains of collagen type I, COL1A1 and COL1A2, mRNA in OA individuals. The exact physiological function of both OCN and ALP is still unknown. OCN, the most abundant non-collagenous protein of the bone extracellular matrix, is synthesised only by bone-forming OB cells. OCN is suggested to be involved in the mineralisation process of newly synthesised osteoid [33]. It is incorporated into the bone matrix, where it is involved in calcium-binding [34]. Quantitative bone histomorphometry and combined calcium balance/calcium kinetics studies have validated the use of OCN as a marker of bone formation [35,36]. Previous studies report increased OCN levels in OA serum, protein, and mRNA gene studies [14,16]. Our finding of differential OCN mRNA gene expression between OA and non-OA individuals is consistent with our previously reported data showing an age-related increase in OCN gene expression in OA and a decrease in controls [16]. This finding is supportive of an increase or maintenance of bone volume in OA individuals versus the age-dependent bone loss in the general population [11]. ALP is used as an enzymatic marker of bone formation, expressed by early-differentiated OB cells. Elevated expression of this enzyme in OA, as indicated by this study as well as previous reports [5,6,15], might indicate a greater proportion of differentiation of the pre-OB pool to the mature phenotype [5].

Even though collagen type I comprises the majority of the organic matrix of bone, it is not unique to the tissue [37]. Thus, non-collagenous proteins such as OCN and OPN have become the focus of studies aimed at the elucidation of the bone matrix mineralisation process in normal and pathological conditions [14,38]. OPN is a cytokine currently understood to be involved in cell attachment, cell migration, chemotaxis, and intracellular signalling and is expressed by all three bone cell types: OB, osteoclasts, and osteocytes [39-41]. In bone, OPN produced by OB during bone matrix formation is subsequently accumulated in the mineralised matrix. Increased OPN may augment OB synthetic activity by increasing OB longevity and surface extent of bone formation. Hence, increased OPN mRNA expression in OA may contribute to the maintenance or increase in bone mass in these individuals.

IGF-I, IGF-II, and TGF-β1 are established osteotropic growth factors that play key roles in bone remodelling and are produced by the various bone marrow and bone cell types in the bone microenvironment [42,43]. The role of these growth factors suggests their involvement in the preservation of the bone matrix. The two related IGFs are involved in inducing matrix apposition and decreasing collagen degradation and expression of interstitial collagenases [44,45]. The main bone anabolic roles for TGF-β1 include stimulating chemotaxis and proliferation to increase the pool of committed OB precursors [46,47]. These growth factors have been reported to be upregulated in OA subchondral and iliac crest bone [6,15,18], and this upregulation is hypothesised to be due to increased OB biosynthetic activity [18]. The lack of differential gene expression of these anabolic growth factors in our study is most likely due to the gene expression being contributed by the various cell types present in the bone microenvironment. In addition to post-transcriptional and post-translational modifications, the well-known regulation of stored growth factors in the extracellular matrix by binding proteins that are released during bone resorption phases may account for the altered protein expression levels in OA *ex vivo *studies. Bone cells produce both IGF-I and IGF-II, and IGF-II is reported to be expressed at higher levels than IGF-I in OA and control subchondral bone [32]. Interestingly, even though IGF-II mRNA expression was significantly higher than IGF-I mRNA in OA, this study indicated similar positive associations between the two IGFs in both OA and non-OA individuals, indicating that the probable co-expression of the two IGFs is not significantly altered at the IT region in hip OA disease. The differential gene expression of ALP, OCN, OPN, and the alpha chains of collagen type I in OA observed from this study suggests support of the hypothesis of increased OB biosynthetic activity, as postulated by Dequeker and colleagues [18]. Further experiments using *in situ *hybridisation and immunohistochemical staining at this distal skeletal site would confirm whether there is an increase in OB biosynthetic activity or increased OB cell number in OA individuals. Additionally, the significant differential gene expression in OA versus control IT trabecular bone may be due to the presence of an altered bone cell phenotype [19].

The trabecular bone samples obtained from patients with OA were architecturally distinct, having elevated bone surface density and Tb.N and decreased Tb.Sp compared to the age- and gender-matched controls. These observations are consistent with previous studies at the same distal skeletal site [12,24] and indicate a more generalised distribution of OA bone architectural changes in OA individuals. In contrast to Fazzalari and colleagues [24], we did not observe age-related changes in bone volume fraction in controls, due to the fact that the aforementioned study analysed a broader age range of subjects. Our control data indicated a significant increase in bone surface density and a significant decrease in Tb.Th with age.

Subchondral bone as well as cancellous bone from the central regions of the femoral head and femoral neck of patients with late-stage OA has been described as hypomineralised [4,6,7], which may be due in part to increased bone remodelling due to adaptation/repair of the diseased joint and therefore does not allow sufficient time for complete mineralisation. In this study of the IT region of the femur, however, the static histomorphometric indices for bone formation and bone resorption were similar in magnitude between the OA and control groups, and the data further indicated that the bone turnover process remains coupled in both groups. These results are in contrast to our previous study of a different OA and control sample set of the IT region, in which we reported increased percentage of bone forming surface for any given amount of bone resorption in OA compared to a non-OA group [24]. The finding of altered bone remodelling in OA [24], however, was confirmed when the data set for OS/BS and ES/BS from Fazzalari and colleagues [24] and the data set from the current study were combined (data not shown). Dynamic histoquantitation of bone remodelling would provide a more comprehensive insight into the rate of turnover at this distal skeletal site.

Bailey and colleagues [8] have identified the presence of collagen type I homotrimer in OA subchondral bone which consists of three α1 chains instead of the usual α1/α2 chain ratio of 2:1. By means of electron microscopy, the collagen fibres were observed to be narrower and aligned in a disorganised manner, which may contribute to the under-mineralisation in OA [8]. Interestingly, the findings of the present study indicated that the ratio of COL1A1/COL1A2 mRNA expression was significantly elevated in OA bone compared to control, suggesting the possible presence of collagen type I homotrimer at a skeletal site distal to the articular cartilage. However, protein analysis determining the expression level of the two alpha chains in the bone matrix at the IT region as well as mineral density fractionation studies will be required to support this notion.

Our experimental approach of combining gene expression analysis with histomorphometry allows the exploration of any relationships between gene expression and indices of bone architecture and bone remodelling. Interestingly, both OCN and ALP gene expression significantly correlated with bone micro-architecture at this distal skeletal site. In controls, there is increased bone volume fraction, bone surface density, and Tb.N, decreased Tb.Sp with increasing OCN mRNA, and an apparent inverse involvement for ALP mRNA, with negative associations with bone volume, bone surface density, and Tb.N. Indeed, from our results, when OCN and bone volume fraction were plotted for the controls, there was a significant increase in bone volume with increasing OCN mRNA levels, which is contradictory to the findings of the OCN knockout mice experiments [48], which suggest that OCN limits bone formation without directly impairing bone resorption or mineralisation. We can speculate that the results of our study may reflect regulatory mechanisms of bone formation in the normal bone remodelling process that are dysregulated in the skeleton of patients with primary hip OA. The pooled OA and control data suggest that there is a continuum in the bone remodelling process leading to particular trabecular structures, with respect to OCN/GAPDH mRNA levels in the IT region. However, the lack of association observed between OCN mRNA expression and the architectural parameters in the OA group may be due in part to altered bone cell response indicated by an increased level of OCN gene expression in OA. The altered cellular response may manifest as an increased range of Tb.N for OCN gene expression levels in OA when compared to controls. On the other hand, Tb.Sp appeared to plateau in the amount of Tb.Sp change, with respect to OCN/GAPDH mRNA levels in the OA group. This is consistent with the reported observation that changes in trabecular architecture are non-linear [49]. Consequently, Tb.Sp is non-linearly associated with OCN/GAPDH mRNA gene expression. The data in this study also indicated that with increasing ALP gene expression there is decreasing bone volume, also inconsistent with ALP knockout studies that have found decreased bone volume, hypomineralisation, and disordered mineral crystal alignment pattern and matrix architecture in metaphyseal bone trabeculae and cortical bone of mice femora and upper tibias [50,51]. Our observations may implicate an increase in bone turnover with increasing ALP gene expression and, thus, a decrease in bone volume due to decreasing Tb.N. Taken together, the observations from these association analyses suggest that any potential regulatory mechanisms between either OCN or ALP with bone architectural parameters are clearly altered in individuals with primary hip OA compared to non-OA controls.

The significant gene expression and bone architectural changes observed between primary hip OA compared to non-OA controls at a skeletal site distal to the active site of disease support the concept that there is a generalised involvement of bone in the pathogenesis of OA. This study, using molecular histomorphometry, has also provided unique insight into possible perturbations or dysregulation of bone turnover processes in OA which differ from the norm. The changes present at distal skeletal sites may be reflective of constitutive gene expression and consequent skeletal structure that predisposes an individual to primary OA. Pre-existing genetic differences in individuals which lead to altered suboptimal loading bone structures that adapt differently to the loads the skeleton is subjected to may affect biomechanical and biochemical profiles, as well as accumulation of microdamage [52], and enhance the initiation/progression of OA. Future research investigating early OA and the diseased joint as a whole is important to further the understanding of OA pathogenesis and, hence, enable development of improved treatment and/or preventative measures to delay joint degeneration or progression of OA.

## Conclusion

Expression of mRNA corresponding to ALP, OCN, OPN, and the two collagen type I alpha chains, COL1A1 and COL1A2, is elevated in primary hip OA IT bone compared to postmortem non-OA controls. The finding of differential gene expression, as well as architectural changes and differences in molecular histomorphometric associations between OA and controls, at a skeletal site distal to the active joint cartilage degeneration supports the concept of generalised involvement of bone in the pathogenesis of OA.

## Abbreviations

ALP = alkaline phosphatase; BMD = bone mineral density; BS/BV = specific surface of bone; BS/TV = bone surface density; BV/TV = bone tissue volume; COL1A = collagen type I alpha chain; ES/BS = eroded surface; GAPDH = glyceraldehyde-3-phosphate dehydrogenase; IGF = insulin-like growth factor; IT = intertrochanteric; OA = osteoarthritis; OB = osteoblast; OCN = osteocalcin; OPN = osteopontin; OS/BS = osteoid surface; SD = standard deviation; SQRT-PCR = semi-quantitative reverse transcription-polymerase chain reaction; Tb.N = trabecular number; Tb.Sp = trabecular separation; Tb.Th = trabecular thickness; TGF-β1 = transforming growth factor-β1.

## Competing interests

The authors declare that they have no competing interests.

## Authors' contributions

JSK and NLF contributed to the study design and coordination. LT performed the acquisition of the SQRT-PCR data. JSK and HT performed the acquisition of the histomorphometry data. LT and JSK performed the statistical analyses. LT, JSK, and NLF analysed and interpreted the data. LT, JSK, and NLF prepared the manuscript. All authors read and approved the final manuscript.

## Figures and Tables

**Table 1:** Primer design for semi-quantitative reverse transcription-polymerase chain reaction analysis

Target gene	Primer sequence (5'-3')	PCR product size (bp)	Annealing temperature (°C)	Number of PCR cycles	GenBank accession number
*ALP*					
Sense	ccaacgtggctaagaatgTC	434	58	30	NM_000478
Antisense	catctcgttgtctgagtacc				
*OCN*					
Sense	ggtgcagcctttgtgtccaagc	159	62	29	NM_199173
Antisense	GTCAGCCAACTCGTCACAGTCC				
*OPN*					
Sense	AGCCGTGGGAAGGACAGTTATG	472	62	29	NM_000582
Antisense	GAGTTTCCATGAAGCCACAAAC				
*IGF-I*					
Sense	GAGCCTGCGCAATGGAATAAAG	344	62	33	NM_000618
Antisense	CCTGTCTCCACACACGAACTG				
*IGF-II*					
Sense	GAGGAGTGCTGTTTCCGCAG	263	62	27	NM_000612
Antisense	ACGTTTGGCCTCCCTGAACG				
*TGF-β1 *[53]					
Sense	CTAGACCCTTTCTCCTCCAGGAGACG	224	62	26	NM_000660
Antisense	GCTGGGGGTCTCCCGGCAAAAGGT				
*COL1A1*					
Sense	CGGCAAGGTGTTGTGCGATG	339	62	33	NM_000088
Antisense	CACGGAAATTCCTCCGGTTG				
*COL1A2*					
Sense	CGCTGGTGAAGTTGGCAAACCA	778	66	31	NM_000089
Antisense	GAGGACCACGAAGCCCTTCTTTC				
*GAPDH *[16]					
Sense	CATGGAGAAGGCTGGGGCTC	415	62	23	NM_002046
Antisense	CACTGACACGTTGGCAGTGG				

ALP, alkaline phosphatase; COL1A, collagen type I alpha chain; GAPDH, glyceraldehyde-3-phosphate dehydrogenase; IGF, insulin-like growth factor; OCN, osteocalcin; OPN, osteopontin; PCR, polymerase chain reaction; TGF-β1, transforming growth factor-β1.

**Table 2:** Semi-quantitative reverse transcription-polymerase chain reaction product/GAPDH ratios for OA and control individuals

Ratio	OA	Control
	(*n *= 15)	(*n *= 13)
IGF-I/GAPDH	0.52 (0.49–0.56)^a^	0.63 (0.38–0.71)
IGF-II/GAPDH	0.79 (0.74–0.94)^a^	0.58 (0.39–0.94)
TGF-β1/GAPDH	0.76 ± 0.22	0.72 ± 0.09
COL1A1/GAPDH	0.55 (0.47–0.59)	0.00 (0.00–0.24)^b^
COL1A2/GAPDH	0.37 ± 0.04	0.22 ± 0.14^c^

^a^OA IGF-I/GAPDH versus OA IGF-II/GAPDH: *p *< 0.0003; ^b^*p *< 0.0001; ^c^*p *< 0.002. Parametric values are mean ± standard deviation. Non-parametric values are median (quartiles). COL1A, collagen type I alpha chain; GAPDH, glyceraldehyde-3-phosphate dehydrogenase; IGF, insulin-like growth factor; OA, osteoarthritis; TGF-β1, transforming growth factor-β1.

**Table 3:** Trabecular bone structure and bone turnover indices in osteoarthritis and control intertrochanteric bone samples

Histomorphometric parameter	Osteoarthritis	Control
	(*n *= 14)	(*n *= 13)
BV/TV (percentage)	10.4 ± 4.1	7.6 ± 3.1
BS/TV (mm^2^/mm^3^)	2.3 ± 0.7	1.3 ± 0.4^a^
BS/BV (mm^2^/mm^3^)	20.5 (17.3–28.6)	18.0 (14.7–20.6)
Tb.Th (μm)	98 ± 39	111 ± 34
Tb.Sp (μm)	817 (760–892)	1,406 (1,203–2,202)^b^
Tb.N (number/mm)	1.11 ± 0.36	0.63 ± 0.19^a^
OV/TV (percentage)	0.12 (0.07–0.15)	0.05 (0.04–0.13)
OS/BS (percentage)	7.8 (3.7–9.7)	7.2 (4.2–9.7)
ES/BS (percentage)	4.6 (3.4–6.4)	4.5 (2.0–7.6)

^a^*p *< 0.0003; ^b^*p *< 0.0001. Parametric values are mean ± standard deviation. Non-parametric values are median (quartiles). BS/BV, specific surface of bone; BS/TV, bone surface density; BV/TV, bone tissue volume; ES/BS, eroded surface; OS/BS, osteoid surface; OV/TV, osteoid volume; Tb.N, trabecular number; Tb.Sp, trabecular separation; Tb.Th, trabecular thickness.
